# Anterior thalamic nucleus inputs to the retrosplenial cortex are unperturbed in the hAPP-J20 mouse model of Alzheimer’s disease

**DOI:** 10.1093/braincomms/fcag417

**Published:** 2026-10-30

**Authors:** Gabriella Margetts-Smith, Lilya Andrianova, Alissa K Plant, Shivali Kohli, Cassandra Sampaio-Baptista, Andrew D Randall, John P Aggleton, Jonathan Witton, Michael T Craig

**Affiliations:** Institute of Biomedical and Clinical Science, University of Exeter, Exeter EX4 4PS, UK; School of Physiology, Pharmacology and Neuroscience, University of Bristol, Bristol BS8 1TD, UK; Institute of Biomedical and Clinical Science, University of Exeter, Exeter EX4 4PS, UK; School of Psychology & Neuroscience, College of Medical, Veterinary and Life Sciences, University of Glasgow, Glasgow G12 8QQ, UK; School of Psychology & Neuroscience, College of Medical, Veterinary and Life Sciences, University of Glasgow, Glasgow G12 8QQ, UK; Institute of Biomedical and Clinical Science, University of Exeter, Exeter EX4 4PS, UK; School of Psychology & Neuroscience, College of Medical, Veterinary and Life Sciences, University of Glasgow, Glasgow G12 8QQ, UK; Institute of Biomedical and Clinical Science, University of Exeter, Exeter EX4 4PS, UK; School of Physiology, Pharmacology and Neuroscience, University of Bristol, Bristol BS8 1TD, UK; School of Psychology, Cardiff University, Cardiff, Wales CF10 3AT, UK; Institute of Biomedical and Clinical Science, University of Exeter, Exeter EX4 4PS, UK; Institute of Biomedical and Clinical Science, University of Exeter, Exeter EX4 4PS, UK; School of Psychology & Neuroscience, College of Medical, Veterinary and Life Sciences, University of Glasgow, Glasgow G12 8QQ, UK

**Keywords:** retrosplenial cortex, anterior thalamic nuclei, electrophysiology, amyloidopathy, dementia

## Abstract

Alzheimer’s disease is a neurodegenerative condition characterized by progressive loss of memory and general decline in cognitive function. Although research has traditionally focused on the hippocampus and entorhinal cortex, other regions important for memory and spatial navigation, such as the anterior thalamic nuclei and retrosplenial cortex, are also affected. The retrosplenial cortex is an important brain region for both memory and spatial navigation, and displays dysfunction in humans during prodromal phases of Alzheimer’s Disease. The anterior thalamic nuclei provide a major input to the retrosplenial cortex, with loss of this input to the retrosplenial cortex causing a dramatic reduction in the expression of the immediate-early gene product c-Fos. The anterior thalamic nuclei to retrosplenial cortex connection may thus present a new target for therapeutic intervention in Alzheimer’s Disease. Here, we set out to determine whether Alzheimer’s-like pathology perturbed projections from the anterior thalamic nuclei to retrosplenial cortex in the hAPP-J20 amyloidopathy mouse model, using optogenetic activation of thalamic inputs combined with *ex vivo* patch clamp electrophysiology. We found that the retrosplenial cortex displayed an age-dependent decline in basal c-Fos activity, with no additional effect of amyloid pathology on c-Fos expression. At all-time points (3, 6 and 9 months) measured, we found no evidence of impairments in the synaptic strength or efficacy of the anterior thalamic nuclei projections to either granular or dysgranular subdivisions of the retrosplenial cortex. We conclude that, in the hAPP-J20 mouse model, this pathway is unaffected by amyloid-β overexpression.

## Teaser

Margetts-Smith *et al*. assessed anterior thalamic nucleus inputs to retrosplenial cortex in the hAPP-J20 Alzheimer’s model using *ex vivo* electrophysiology. Despite amyloid deposition and age-related declines in c-Fos activity, synaptic strength and connectivity of this pathway remained intact. These findings suggest that thalamo-retrosplenial signalling may be resilient in early-stage amyloidopathy.

## Graphical Abstract

**Graphical Abstract:**
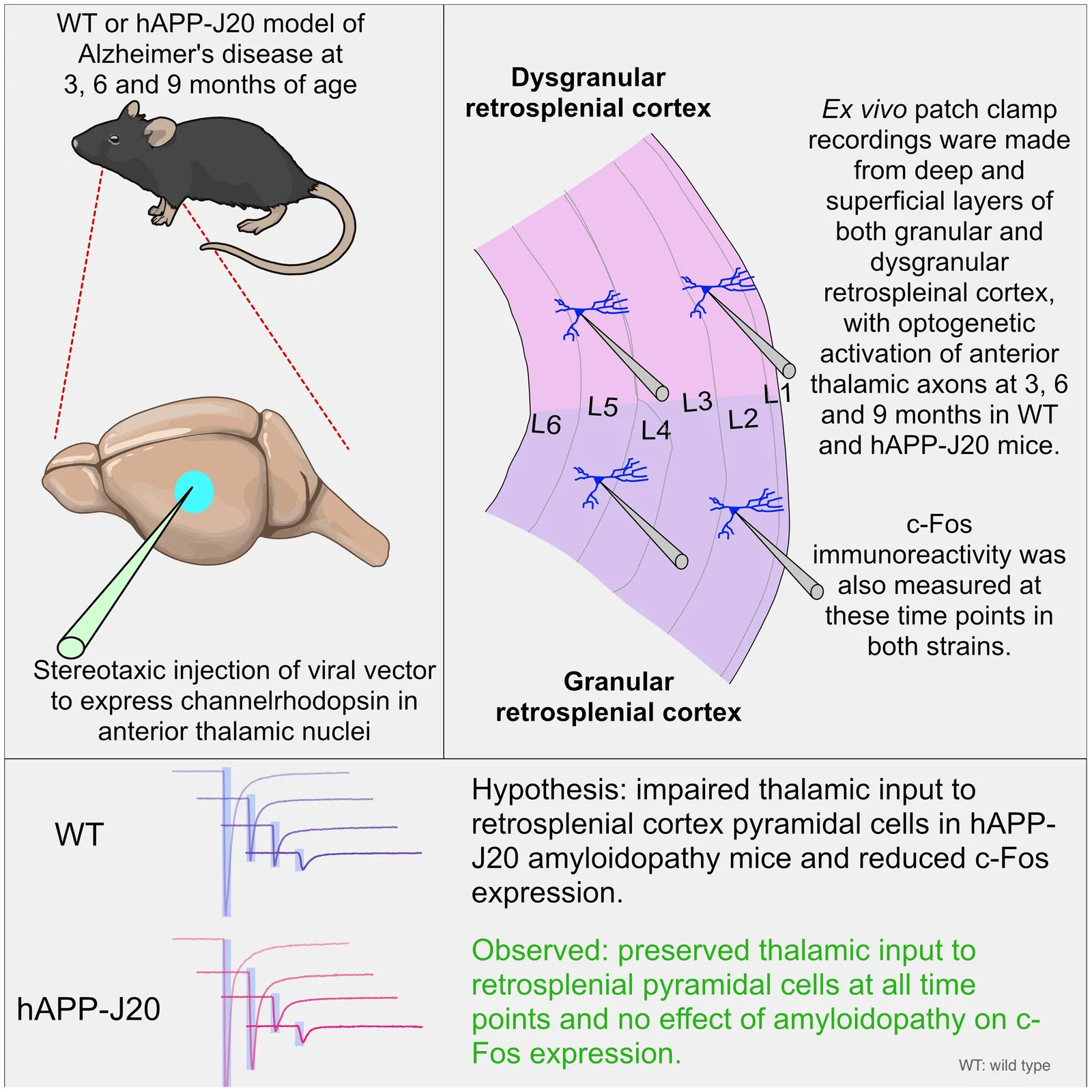

## Introduction

Alzheimer’s disease (AD) is a progressive neurodegenerative disease that typically initiates in entorhinal and hippocampal regions^1^ and also specifically affects the midline and anterior thalamic nuclei (ATN) that project to these regions.^2,3^ Impairments in memory are often the first symptoms that present in individuals living with Alzheimer’s disease,^4^ although the consensus is that pathology may be present for 10 to 20 years before the onset of symptoms.^5^ Understanding how AD affects the neural circuitry underlying memory at different points in disease progression is an essential first step to developing targeted, circuits-based treatments to allow amelioration of memory deficits once the disease has begun to progress. While research into the neurobiology of memory has typically focused on the hippocampus, there are numerous other, equally important brain regions such as the entorhinal and retrosplenial cortices, that are involved in the formation and maintenance of memory. Alongside the midline thalamus, the ATN play a key role in serving as a hub for communication throughout the brain’s extended memory and present an attractive target for therapeutic intervention into spatial and episodic memory disorders.^6^

One brain region of particular interest in the extended memory circuit is the retrosplenial cortex (RSC). The retrosplenial cortex is highly interconnected with hippocampal, parahippocampal and anterior thalamic regions, with this connectivity highly conserved across mammalian species.^7^ The RSC also receives input from sensory regions such as visual cortex and is believed to have important roles in both memory and spatial navigation.^7^ In humans, the RSC is a site of reduced metabolism in the early stages of AD,^8^ with impaired functional connectivity sufficient to distinguish RSC from posterior cingulate cortex in prodromal AD.^9^ Other studies have found that prodromal AD is associated with RSC hypometabolism, atrophy, synapse loss and Aβ accumulation.^10-13^ A recent combined human and rodent study found dysregulation of the RSC in AD was associated with impairments in the function of inhibitory GABAergic neurons.^14^ This latter study highlights the need for animal studies to better understand the function of RSC in health and disease, given the difficulty of directly manipulating RSC in humans.^3,7^

In mouse models of amyloidopathy, the RSC exhibits significant Aβ deposition that is concurrent with neuroinflammatory responses as well as cognitive impairment in the 5xFAD model.^15^ The RSC also displays decreased neuronal activity in response to a novel environment at a pre-clinical stage preceding plaque deposition in the Tg2576 amyloidopathy model.^16^ Indeed, the RSC is sensitive to loss of synaptic inputs, with deafferentation via hippocampal^17^ or anterior thalamic nuclear lesions leading to a dramatic reduction in the expression of the immediate-early gene product c-Fos.

Altogether, it is clear that the RSC may be important both in pathophysiology of Alzheimer’s disease as well as presenting a tempting therapeutic target. In the present study, we used the hAPP-J20 amyloidogenic mouse model^18^ of AD to determine whether markers of activity in RSC were impaired at time points relating to pre-symptomatic, prodromal and symptomatic phases of the disorder. By 9 months of age, hAPP-J20 mice display impairments in spatial memory,^19^ and deficits in novelty-induced beta oscillations in RSC,^20^ making this line ideal for our study. We also used optogenetic activation of ATN inputs to the RSC, combined with patch-clamp electrophysiology, to test the hypothesis that the ATN to RSC projection would specifically be impaired in this mouse model.

## Materials and methods

### Animals

All experiments were conducted in accordance with the UK Animals (Scientific Procedures) Act 1986 under Project licence PAC082CD3, after local ethical review by the Animal Welfare and approved by the University of Exeter Institutional Ethical Review Board. All animals were maintained on a 12 h constant light/dark cycle and procedures and experiments were conducted during the light phase. All animals had access to food and water *ad libitum* and were group-housed wherever possible. We used standard home-cage enrichment that included cardboard tubes and domes, wooden chew blocks and nesting material.

For immunohistochemistry experiments, we used 49 wild-type (WT) and heterozygous transgenic hAPP-J20^18^ mice. Founder mice for our hAPP-J20 colony were kindly supplied by Dr Keith Phillips of Eli Lilly (Erl Wood, Surrey, UK) on a C57BL/6J background and were occasionally back-crossed with C57BL/6J mice to maintain the background. We used heterozygous and WT littermates that were bred from wildtype × heterozygote pairings.

### Drugs and chemicals

CGP55845, Gabazine (SR 95531) and DNQX were purchased from HelloBio (UK), and all other chemicals were purchased from Sigma-Aldrich (UK) unless otherwise stated.

### Immunohistochemistry

For immunohistochemistry experiments, we used 49 wild-type (WT) and hAPP-J20 mice at 3 months (3m), 6 months (6m) and 9 months (9m). 24 WT and 25 hAPP-J20 mice were deeply anaesthetised with sodium pentobarbitone (200 mg/kg; Henry Schein) before being transcardially perfused (5 mL/min) with ice-cold phosphate buffered saline (PBS) for 5 min followed by 4% paraformaldehyde (PFA) in PBS for 10 min. The animals were not handled or exposed to any other non-home cage stimulation for 2 h prior to perfusion in order to capture only basal c-Fos expression. For the c-Fos experiment, mice were group-housed (2 to 5 mice per cage) in GM500 individually vented cages (Techniplast, UK) with dimensions of 399 × 199 × 169 mm. The cages had standard enrichment of cardboard tubes, mouse shelter and bedding. Following the perfusion, the brains were removed and stored in 4% PFA in PBS for 22 h at 4°C, then cryoprotected in 30% sucrose (in PBS with 0.02% Sodium Azide) for at least 3 days. Mice were genotyped prior to perfusion as part of general animal husbandry practices, and genotype was confirmed from tissue removed from the tail after anaesthesia was applied. 30 µm coronal sections were collected and stained for Fos and Aβ plaques, and tissue and staining quality was assessed from Fos-stained sections. Mice were excluded from analysis of Fos and Aβ deposition if sections were highly damaged or if the Fos staining failed (assessed from an absence of staining in the olfactory bulb, piriform cortex, somatosensory cortex and visual cortex). Nine mice were excluded due to low tissue quality, leaving 18 WT (3m: *n*_male_ = 4, *n*_female_ = 5; 6m: *n*_male_ = 1, *n*_female_ = 3; 9m: *n*_male_ = 4, *n*_female_ = 1) and 22 Tg mice (3m: *n*_male_ = 2, *n*_female_ = 5; 6m: *n*_male_ = 4, *n*_female_ = 3; 9m: *n*_male_ = 5, *n*_female_ = 3).

Using a freezing sledge microtome (Leica SM2010R with Physitemp BFS-5MP temperature controller) coronal sections were taken from frozen brains (−20°C). Free-floating sections were stored in PBS containing (0.02%) NaAzide at 4°C or a cryoprotectant solution (25% glycerol, 30% ethylene glycol, 25% 0.2 M Phosphate buffer, 20% ddH_2_O) at −20°C for long-term storage. For immunohistochemistry staining, all steps were conducted at RT unless otherwise stated.

For c-Fos staining, 30 µm free-floating sections stored in PBS-Azide were washed in PBS (3× 10 min), before being incubated in PBS containing 0.09% hydrogen peroxide for 20 min to quench endogenous peroxidase. Next, sections were washed in PBS (3× 10 min), then blocked and permeabilised in PBS-(0.2%)Tx (PBS, 0.2% Triton X-100) containing 3% normal goat serum (NGS). The sections were then incubated in 1:800 anti-Fos primary antibody in PBS-(0.2%)Tx with 3% NGS at 4°C overnight. The following day the sections were washed in PBS (3× 10 min), then incubated for 2 h in 1:600 biotinylated anti-rabbit secondary antibody in PBS-(0.2%)Tx with 1% NGS. Sections were washed in PBS (2× 10 min), then incubated in an Avidin-Biotin complex (ABC) solution for 1 h. After a further three washes in PBS the sections were incubated with 0.04% 3,3′-Diaminobenzidine-tetrahydrochloride (DAB) with 0.04% hydrogen peroxide and 0.05% ammonium nickel(II) sulphate in PBS for ∼10 min. After a final two 10-min washes in PBS, sections were mounted on Superfrost Plus slides and left to dry overnight. The next day, sections were serially dehydrated in graded ethanol (EtOH) baths (all 2 min: 2× ddH_2_O, 30% EtOH, 60% EtOH, 90% EtOH, 95% EtOH, 2× 100% EtOH) and then cleared in Histo-Clear II (2× 10 min). Slides were then sealed and cover slipped using Histo-Mount mounting medium.

The RSC, CA1 region of the hippocampus (HPC) and the entorhinal cortex (EC) were visualized using bright-field microscopy and images captured using a 10× objective on a Nikon Eclipse 800 microscope attached to a SPOT RT monochrome camera running SPOT Basic imaging capture software (SPOT Imaging). Four replicates of each ROI were captured for each mouse, taken from the left and right hemispheres of two sections. Image analysis consisted of an automatic count of nuclei expressing high levels of c-Fos (Fos+) in predefined regions of interest (ROI) using Paxinos & Franklin boundaries^21^ on Fiji^22^ software. To perform this count, images were submitted to a fast Fourier transform bandpass filter and colour scale inversion before being run through the 3D object counter plugin with a brightness threshold (set to 1.7) that depended on average pixel brightness of the filtered image.^23^ Fos^+^ cell counts were then normalized to ROI area, described as Fos^+/mm2^.

For amyloid plaque staining, 30 µm free-floating sections stored in cryoprotectant solution were washed in PBS (3× 10 min) before being mounted onto Superfrost Plus slides and left to dry overnight. The following morning a hydrophobic barrier was drawn around the sections and sections were covered in 70% ethanol for 5 min before being washed with ddH_2_O (2× 2 min). Next, sections were incubated in 1:100 Amylo-Glo^24^ (2B Scientific) solutions in 0.9% saline for 10 min. Sections were then rinsed in 0.9% saline for 5 min, followed by PBS washes (3× 10 min). Sections were left to dry, then cover slipped with Fluoromount mounting medium (Merck Life Sciences).

In a second experiment to confirm amyloid plaque density and number, we instead used immunohistochemistry to visualize plaques. We used the above protocol with the following changes: our primary antibody was Amyloid-β_1–42_ (D9A3A) Rabbit mAb (1:1000, Cell Signalling Technology, #14974), incubated in 1% NGS in PBST at 4°C overnight. The following day, sections were washed in PBS (3× 10 min) before being incubated in Goat-anti-rabbit IgG (H + L) cross-adsorbed secondary antibody, conjugated to Alexa-Fluor 555 (1:200, Thermo Fisher, #A21428) in 1% NGS in PBST for 2 h at room temperature. After a further 3× 10 min washes in PBS, sections were mounted on Superfrost plus slide, sealed and cover slipped using Vectashield Hardset antifade mounting medium (Vector Laboratories, H-1400).

*Amylo-Glo*: Aβ plaques in the RSC, CA1 and EC were visualized using epifluorescent microscopy (Nikon Eclipse 800 microscope with CoolLED pE-4000 LED light source) at 365 nm excitation. Images were captured at 4× magnification using a SPOT RT camera running SPOT Basic imaging capture software. Four replicates of each ROI were captured for each mouse. Anti-Aβ_1–42_: The RSC, dorsal hippocampus and ATN were visualized using an EVOS M7000 microscope at 20×. Six replicates were taken for each ROI for each mouse, taken from the left and right hemisphere of three sections.

Image analysis was conducted using Fiji software; amyloid plaques were quantified by measuring the area covered by plaques in predefined brain ROIs. Plaque areas were manually drawn and measured where present, then normalized as a percentage of brain area, described as Plaque/Area (%).

### Stereotaxic injection surgical procedure

All surgeries were conducted using aseptic technique. Mice were anaesthetized with isoflurane (5% induction, 1.2–2.5% maintenance) delivered in a constant flow of oxygen. The mice were placed on a heated pad (37°C) for the duration of the surgery and given 0.03 mg/kg of buprenorphine (buprenorphine hydrochloride, Henry Schein) subcutaneously at the beginning of surgery as an adjunct analgesic, plus 5 mg/kg of carprofen (Rimadyl, Henry Schein) and 10 mL/kg of 0.9% saline were given subcutaneously immediately post-surgery. 5 mg/kg of carprofen was also provided daily for 3 days post-surgery.

Mice received a unilateral injection into the ATN (AD/AV border; AP: −0.71 mm; ML: ±0.80 mm; DV −2.76 mm from pia) of 250 nL of AAV_5_-hSyn1-ChR2(H134R)-mCherry-WPRE-hGHp(A) (ETH Zurich Viral Vector Facility, titre 7.1 × 10^12^ vg/mL) at a flow rate of 50 nL/min. After the surgery, the mice were allowed at a 4–7 week recovery period to allow sufficient time for the expression of the viral construct, with electrophysiological recordings carried out in mice aged 3, 6, or 9 months.

### Slice preparation and electrophysiology

Mice were anaesthetised with 5% isoflurane and decapitated before the brain was rapidly removed and placed in room temperature (RT) oxygenated *N*-methyl-d-glucamine (NMDG) solution^25^ consisting of (in mM): 135 NMDG, 10 D-Glucose, 1.5 MgCl_2_, 0.5 CaCl_2_, 1 KCl, 1.2 KH_2_PO_4_, 20 Choline bicarbonate. 300 µm coronal slices corresponding to approximately Bregma −0.5 mm to −4.5 mm were sectioned using a Leica VT1200 vibratome. Following sectioning, slices were transferred to a holding chamber containing artificial cerebral spinal fluid (aCSF) perfused with a continuous flow of carbogen (95% O_2_, 5% CO_2_). The aCSF solution consisted of (in mM): 119 NaCl, 3 KCl, 1 NaH_2_PO_4_, 26 NaHCO_3_, 10 D-Glucose, 2.5 CaCl_2_, 1.3 MgCl (pH 7.4). The slices were then incubated at 35°C for 30 min, then at RT for a further 30 min to recover before recording. ATN injection site fluorescence was visually confirmed immediately following slicing, and slices were discarded if viral expression was absent or in an anatomically-incorrect location (*n* = 24 mice). Slices from a total of 68 mice were used: 39 WT (3m: *n*_male_ = 8, *n*_female­_ = 3; 6m: *n*_male_ = 7, *n*_female_ = 9; 9m: *n*_male_ = 5, *n*_female_ = 7) and 29 Tg (3m: *n*_male_ = 4, *n*_female_ = 4; 6m: *n*_male_ = 5, *n*_female_ = 6; 9m: *n*_male_ = 5, *n*_female_ = 5).

RSC containing slices were attached to a 0.1% poly-L-lysine coated coverslip and transferred to the recording chamber where they were perfused with carbogen-saturated aCSF (4 mL/min, 32–34°C). Differential interference contrast (DIC) and fluorescent signal imaging was performed using an Olympus BX51W1 microscope and SciCam Pro camera with a CoolLED pE-4000 LED light source. Borosilicate glass microelectrodes (OD 1.5 mm, ID 0.86 mm, 3–6 MΩ) were fabricated using a P-97 Flaming Brown micropipette puller and filled with caesium methanesulphonate (CsMeSO_4_) intracellular solution containing (in mM): 135 CsMeSO_4_, 8 KCl, 0.5 EGTA, 10 HEPES, 0.5 QX-314, 0.1 Spermine, 0.3 Na_2_-GTP, 2 Mg-ATP. Putative pyramidal cells (PC) were identified under DIC visualization and assigned as being located in the superficial (L2-4) or deep (L5-6) layers of the dRSC or gRSC sub-regions of the RSC dependent on surrounding cytoarchitecture.

Data were collected with a Multiclamp 700B amplifier combined with a Digidata 1440A analogue-to-digital converter and a standalone computer equipped with pClamp software (Molecular Devices). Signals were digitized at 20 kHz and lowpass filtered at 8 kHz. Synaptic properties were recorded in voltage clamp mode, and cells were recorded from at a holding membrane potential (V_H_) of −70 mV and in standard aCSF unless otherwise stated. All optic stimulation was carried out at a 470 nm wavelength generated by a CoolLED pe-4000 light source at 100% power.

Cell response and EPSC magnitude were measured using a stimulation train protocol consisting of a 500 ms −10 mV square voltage injection followed by 5 ms optic stimulation pulses at 7 Hz frequency applied to the cell for 10–20 sweeps. PPR was measured using an increasing inter-pulse interval (IPI) protocol which consisted of: a 500 ms −10 mV square voltage injection followed by two 5 ms optic stimulation pulses at 10, 17, 51, 100, 170, 510 and 1000 ms intervals. Finally, AMPAR- and NMDAR-mediated currents were measured using a protocol consisting of a 500 ms −10 mV square voltage injection followed by a single 5 ms optic stimulation pulse. AMPAR-mediated currents were measured at V_H_ = −70 mV in standard aCSF and NMDAR-mediated currents were measured at V_H_ = +40 mV in aCSF containing antagonists for GABA_A_, GABA_B_ and non-NMDA ionotropic glutamate receptors (standard aCSF containing (in µM): 10 Gabazine, 1 CGP-55845, 10 DNQX).

Custom MATLAB scripts were used to analyse electrophysiological recordings unless stated otherwise. Electrophysiology traces were analysed using Igor Pro 7 (Wavemetrics, OR, USA) or MATLAB (Mathworks, CA, USA) software.

### Statistical analysis

We used R,^26^ implemented in RStudio,^27^ to conduct all statistical analyses and generate all data graphs, using the lme4,^28^ lmerTest,^29^ tidyverse,^30^ ggplot2^31^ and ggpubr packages. For the electrophysiological data in particular, we used mixed effects modelling instead of more standard ANOVA-types of analyses. The reasons for this were 2-fold: firstly, while it is accepted in the field to treat individual neurons from patch clamp experiments as independent samples, this is not the most appropriate from a statistical perspective as there could be variability introduced by slice preparation, viral injection targeting, etc. The mixed effects model allows the individual mouse to be treated as a random effect to take account of this variability. Secondly, our data were highly dimensional (comparing granular versus dysgranular RSC, deep versus superficial, at three different time points and two different genotypes). With ANOVA analyses, the sequence in which variables are introduced to a model affects the output while this does not occur in a mixed effects model, so the mixed effects model was most appropriate for our high-dimension data.

## Results

We first confirmed that heterozygous hAPP-J20 mice showed increased Aβ deposition with age. While no plaques were detectable at 3 months of age using Amylo-Glo,^24^ Aβ expression was present at the other experimental time points, 6 and 9 months in the RSC (Supplementary Fig. 1) and in both the hippocampus and the lateral EC (Supplementary Fig. 2). In a second experiment, we used an anti-Aβ_1–42_ antibody to verify plaque density in ATN, hippocampus and RSC. We found an age-dependent increase in plaque number in the RSC and hippocampus but no evidence of plaque deposition in ATN by 9 months (Supplementary Fig. 3).

### Basal c-Fos reactivity is unchanged in hAPP-J20 mice

c-Fos is a widely-used marker of neuronal activation and it has been reported that its expression in RSC is reduced in the Tg2576 model of amyloidopathy before the onset of plaque deposition.^16^ Furthermore, basal levels of c-Fos have been reported to be reduced in the dentate gyrus of hAPP-J20 mice at 4 to 6 months^32^ despite these mice displaying a hyperexcitability phenotype.^33^ We carried out basal c-Fos staining in the RSC (Fig. 1) as well as the CA1 region of the HPC and the EC (Fig. 2) at 3, 6 and 9 months to determine whether these mice displayed signs of reduced neuronal activation. Sections containing the intermediate to ventral CA1 (AP ∼−2.5 to −3.5) were analysed as this area is known to present a higher amyloid plaque load than the dorsal CA1,^34^ and is known to play a critical role in many mnemonic behaviours disrupted in AD.^35^ Sections from the RSC were selected along the AP axis (∼−1.5 to −4.00), and both pre- and post-splenium sections were included in the analysis for each animal to control for the functional heterogeneity of the area along this axis.^7^ Furthermore, analysis of the RSC ROIs was classified either as the granular or dysgranular sub-regions, as these are also known to exhibit different functionality and connectivity. These basal c-Fos experiments were carried out in the home cage so it should be noted this provides a snapshot of basal neuronal activation, and that we did not measure task-evoked activity.

**Figure 1:**
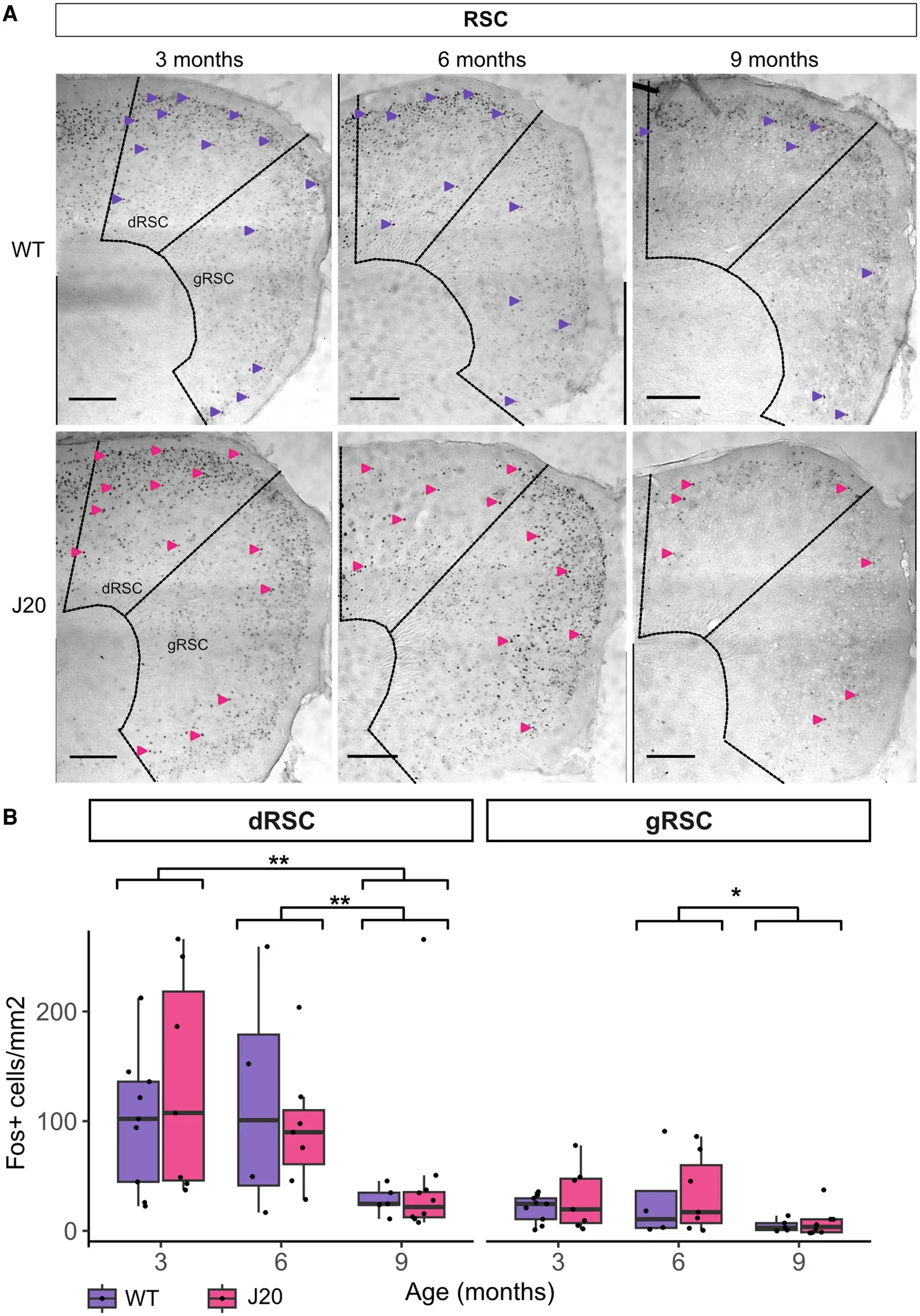
**Basal c-Fos expression differs between RSC sub-regions and decreases with age but is not affected by hAPP-J20 genotype**. (**A**), representative images of basal c-Fos expression in the RSC in WT and Tg hAPP-J20 mice at 3m (WT, *n* = 9; hAPP-J20, *n* = 7), 6m (WT, *n* = 4; hAPP-J20 *n* = 7) and 9m (WT, *n* = 5; hAPP-J20, *n* = 8) age points. Images of the dysgranular RSC (dRSC) and granular RSC (gRSC) were taken separately (10× magnification) and stitched together. Arrows indicate representative cells considered c-Fos^+^ but are not exhaustive of the count. Scale bars: 250 µm. (**B**), a three-way mixed ANOVA indicated a significant main effect of sub-region (*F*(1,34) = 50.82, *P* < 0.001) and age (*F*(2,34) = 6.51, *P* < 0.01), as well as a significant interaction between the two variables (*F*(2,34) = 7.86, *P* < 0.01). *Post hoc* tests comparing age points pooled across genotype in the dRSC found significantly fewer c-Fos^+^ cells/mm^2^ at 9m than 3 and 6m, which did not differ from each other (*P* = 0.65) (3m WT: 100.44 (62.26); 3m Tg: 134.15 (99.37); 6m WT: 119.39 (109.6); 6m Tg: 94.83 (57.47); 9m WT: 27.89 (12.97); 9m Tg: 24.69 (15.32) c-Fos^+^ cells/mm^2^). In the gRSC, there were significantly fewer c-Fos^+^ cells/mm^2^ (pooled across genotype) at 9m than 6m, but not compared with 3m (*P* = 0.07). 3m and 6m also did not significantly differ in Fos expression (*P* = 0.43) (3m WT: 25.88 (12.44); 3m Tg: 35.17 (28.49); 6m WT: 33.73 (42.29); 6m Tg: 39.28 (35.08); 9m WT: 10.21 (5.73); 9m Tg: 12.97 (13.05) c-Fos^+^ cells^mm2^). There was no significant main effect of genotype (*F*(1,34) = 0.08, *P* = 0.79), or interaction between genotype and age (*F*(2,34) = 0.45, *P* = 0.64) or sub-region (*F*(1,34) = 0.06, *P* = 0.81). Finally, there was no significant interaction between all three independent variables (*F*(2,34) = 0.99, *P* = 0.38). Descriptive stats display mean and SD. Boxplots display median (solid line), IQR and range. All pairwise comparisons across age or genotype were non-significant unless otherwise shown; * P < 0.05, ** *P* < 0.01. Each data point represents one mouse, with both males and female mice used (see Materials and methods for full breakdown of sex and age information).

**Figure 2:**
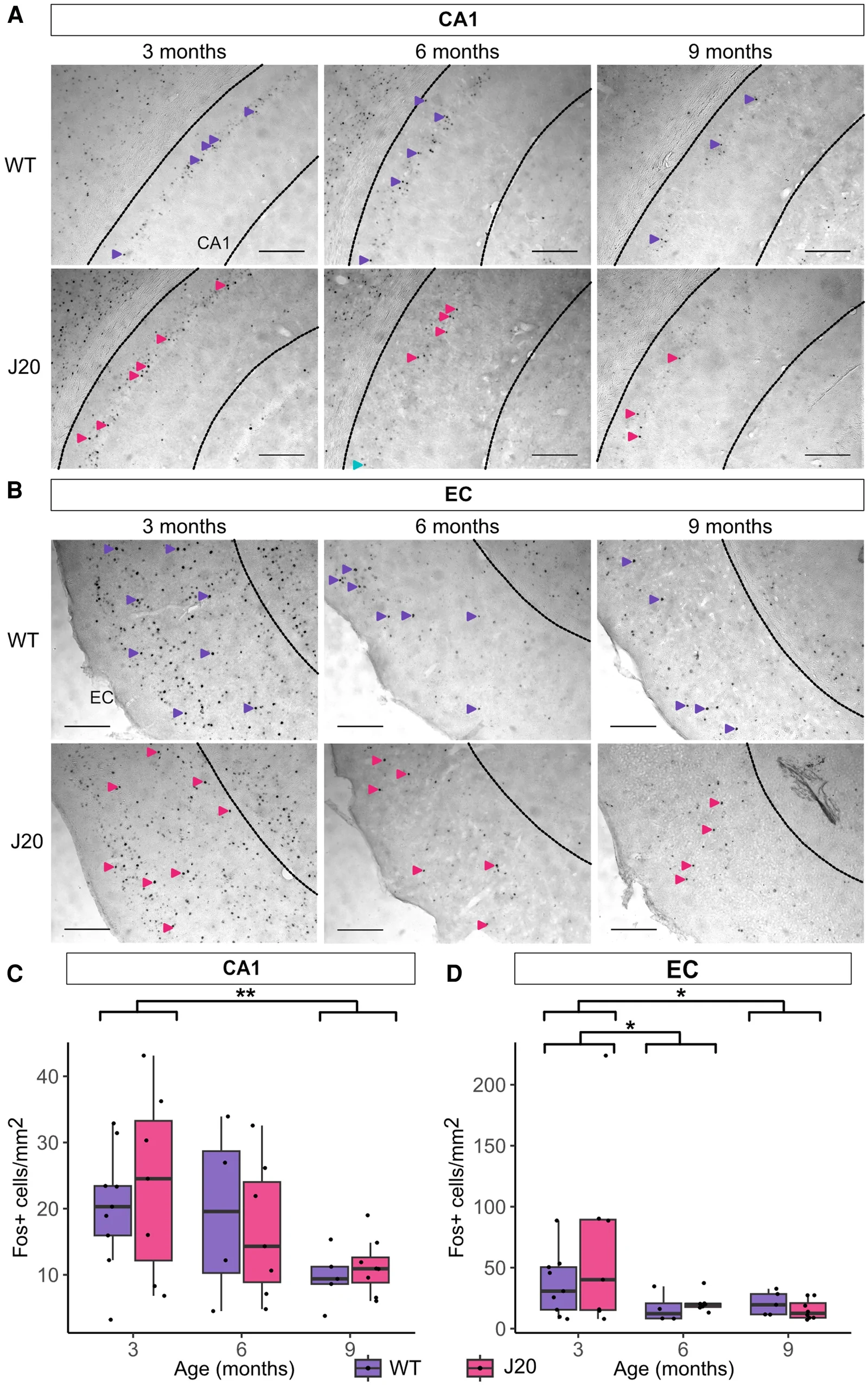
**Basal c-Fos expression in CA1 and EC decreases with age but is not affected by hAPP-J20 genotype**. A-B, Representative images of basal c-Fos expression in the CA1 (**A**) and EC (**B**) in WT and Tg hAPP-J20 mice at 3m, 6m, and 9m age points. Images were taken at 10× magnification, and arrows indicate representative cells considered c-Fos^+^ but are not exhaustive of the count. Scale bars: 250 µm. (**C**), in CA1 there was a main effect of age (*F*(2,34) = 4.75, *P* < 0.05; 2-way ANOVA), but no effect of genotype (*F*(1,34) = 0.002, *P* = 0.96) or interaction between age and genotype (*F*(2,34) = 0.30, *P* = 0.74). *Post hoc* tests found, when pooled across genotype, that c-Fos expression was significantly lower at 9m than 3m, but there was no difference between 6m and 9m (*P* = 0.11) or 3m and 6m (*P* = 0.29) (**3m WT:** 20.18 (9.21); **3m Tg:** 23.62 (13.90); **6m WT:** 19.40 (13.43); **6m Tg:** 16.78 (10.35); **9m WT:** 9.67 (4.19); **9m Tg:** 11.22 (4.24) c-Fos^+^ cells^mm2^). (**D**), in the EC there was a significant main effect of age (*F*(2,34) = 4.46, *P* < 0.05; 2-way ANOVA), but no main effect of genotype (*F*(1,34) = 0.33, *P* = 0.57) or interaction between age and genotype (*F*(2,34) = 0.11, *P* = 0.90). *Post hoc* tests indicated that c-Fos expression, when pooled across genotype, at 3m was significantly higher than 6m or 9m, while expression did not significantly differ between those age points (*P* = 0.89) (**3m WT:** 36.41 (26.00); **3m Tg:** 68.76 (76.55); **6m WT:** 16.85 (12.45); **6m Tg:** 20.98 (7.67); **9m WT:** 20.77 (9.63); **9m Tg:** 15.47 (8.17) c-Fos+ cells/mm^2^). Descriptive stats display mean and SD. Boxplots display median (solid line), IQR and range. All pairwise comparisons across age or genotype were non-significant unless otherwise shown; ** *P* < 0.01, * *P* < 0.05. Each data point represents one mouse, with both males and female mice used (see Materials and methods for full breakdown of sex and age information).

We observed that basal c-Fos expression varied significantly between RSC subdivisions, with the dysgranular subdivision having a significantly higher level of expression than the granular subdivision (Fig. 1B). We found that, while basal c-Fos expression was reduced at 9 months in both RSC subdivisions, there was no effect of genotype at any time point, with hAPP-J20 mice having comparable levels of basal c-Fos expression at 3, 6 and 9 months of age. No amyloid plaques were observed at 3 months but are apparent from 6 months of age in Tg mice, but not WT controls (Supplementary Figs 1 and 3). Furthermore, whilst c-Fos expression decreased with age, there was also a significant interaction between age and sub-region: the dRSC displayed a greater decrease in c-Fos+ cells as age increased. *Post hoc* analyses (Fig. 1B) found no significant differences between WT and Tg mice at any age point in the dRSC (3m WT versus Tg: *P* = 0.42; 6m WT versus Tg: *P* = 0.63; 9m WT versus Tg: *P* = 0.71) or gRSC (3m WT versus Tg: *P* = 0.39; 6m WT versus Tg: *P* = 0.82; 9m WT versus Tg: *P* = 0.67). This suggests that while baseline neuronal activity is significantly decreased by 9 months (with a more pronounced reduction in dRSC with age), amyloidopathy does not alter basal c-Fos expression in this region at prodromal or clinical stages.

To examine whether this lack of effect of amyloidopathy on basal activity was restricted to the RSC, sections containing the CA1 (Fig. 2A) and EC (Fig. 2B) were also quantified and analysed. Both brain regions showed a similar pattern to the dRSC and gRSC in that c-Fos expression decreased with age, but was not affected by genotype (Fig. 2C, D). *Post hoc* analyses in CA1 revealed no significant differences between wild-type and transgenic mice at each time point (3m WT versus Tg: *P* = 0.56; 6m WT versus Tg: *P* = 0.73; 9m WT versus Tg: *P* = 0.53), and the same was true in the EC (3m WT versus Tg: *P* = 0.25; 6m WT versus Tg: *P* = 0.51; 9m WT versus Tg: *P* = 0.31). Overall, we conclude that while amyloid plague deposition is occurring in these mice, and despite our previous observations that deficits in spatial memory occur in this line at 9 months of age,^19^ there was no observable change in basal c-Fos expression in brain regions associated with spatial memory.

### No change in thalamic projections to RSC glutamatergic neurons in hAPP-J20 mice

Deficits in synaptic transmission have been widely reported in hAPP-J20 mouse line^33,36-38^ as well as other amyloidopathy models. We recently reported that the ATN input to RSC is ubiquitous, with all excitatory neurons receiving input.^39^ Given that the ATN have been targeted in attempts to reverse deficits in rodent models of dementia^19^ and other models of memory disorder,^40^ we sought to test the hypothesis that ATN inputs to RSC would show age-dependent impairments in the hAPP-J20 mouse model. To do this, we carried out stereotaxic injections of AAV vectors into WT and hAPP-J20 mice to transduce expression of channelrhodopsin into the ATN and carried out *ex vivo* patch-clamp electrophysiological recordings a 3, 6 and 9 months.

We targeted pyramidal neurons from deep and superficial layers of both dRSC and gRSC, recording from a total of 438 neurons, of which 305 passed quality checks (see Materials and methods), from 68 mice of both sexes. First, we checked for the presence of an EPSC in response to optogenetic stimulation of ATN axons. Like our previous findings in C57BL/6J mice,^39^ all putative pyramidal cells responded to stimulation of this pathway for both genotypes and all age points. Therefore, probability of synaptic connectivity (the percentage of recording neurons responding to optogenetic stimulation of thalamic axons) was not altered by age or genotype. Further analysis of the properties of the ATN-driven EPSC was then carried out (details in Table 1).

**Table 1:** Summary of all recorded and analysed neurons separated by age, genotype, RSC sub-region and cortical layer

				Count		
Age	Genotype	Sub-region	Layer	EPSC measures	NMDA/AMPA	PPR
3m (*n* = 189)	WT (*n* = 100)	dRSC	Superficial	12	6	9
			Deep	12	3	12
		gRSC	Superficial	11	5	7
			Deep	12	2	9
	Tg (*n* = 89)	dRSC	Superficial	10	5	9
			Deep	8	3	7
		gRSC	Superficial	11	5	10
			Deep	12	2	7
6m (*n* = 284)	WT (*n* = 142)	dRSC	Superficial	15	8	10
			Deep	15	2	13
		gRSC	Superficial	19	7	17
			Deep	16	6	14
	Tg (*n* = 142)	dRSC	Superficial	20	10	16
			Deep	13	7	11
		gRSC	Superficial	13	7	11
			Deep	14	8	12
9m (*n* = 208)	WT (*n* = 133)	dRSC	Superficial	13	8	12
			Deep	16	6	13
		gRSC	Superficial	12	6	11
			Deep	15	6	15
	Tg (*n* = 75)	dRSC	Superficial	13	7	8
			Deep	5	1	3
		gRSC	Superficial	12	5	9
			Deep	6	2	4
			Total	305	127	249

All putative RSC pyramidal cells responded to stimulation of ATN afferent terminals. EPSC magnitude, onset time and 20–80% rise time were calculated from 305 neurons total. NMDA/AMPA ratio was calculated from 127 neurons of these cells, and PPR from 249 cells.

To examine the magnitude of the AMPA receptor-mediated component of the ATN-EPSC onto RSC pyramidal cells, a null model (*random effect*: mouse) and three mixed effect models were created. The goodness-of-fit (represented by AIC) was sequentially evaluated using χ^2^ likelihood ratio comparisons. Model 1 (*random effect*: mouse; *fixed effects:* sub-region and layer) significantly improved upon the null model (*χ*^2^(2) = 73.0, *P* < 0.001; AIC_null_ = 4595.0, AIC_M1_ = 4526.0), and both sub-region (*F*(1280.1) = 62.2, *P* < 0.001; ANOVA) and layer (*F*(1286.1) = 286.1, *P* < 0.001; ANOVA) significantly affected EPSC magnitude. Fixed effect estimates (Table 2) indicate that cells in the gRSC and deep layers had a significantly lower EPSC magnitude compared with those in the dRSC and superficial layers, respectively. Inspection of the data shows that cells in the superficial layers of the dRSC had the largest EPSC magnitude, followed by dRSC deep layer cells (Fig. 3) in line with our previous findings.^39^ Next, model 2 added age and genotype as fixed effects (*random effect:* mouse; *fixed effects*: sub-region, layer, age and genotype); however this model did not improve upon model 1 (*χ*^2^(3) = 3.9, *P* = 0.27; AIC_M2_ = 4528.1). Furthermore, the addition of sex as a fixed effect in model 3 (*random effect:* mouse; *fixed effects*: sub-region, layer, age, genotype and sex) did not improve upon model 2 (*χ*^2^(1) = 2.8, *P* = 0.09; AIC_M3_ = 4527.7), and fixed effect estimates for models 2 and 3 did not show any significant differences in EPSC magnitude for these added factors (Table 2). Overall, model 1 was the best model of EPSC magnitude variability, suggesting age, genotype and sex have no effect on this measure.

**Figure 3:**
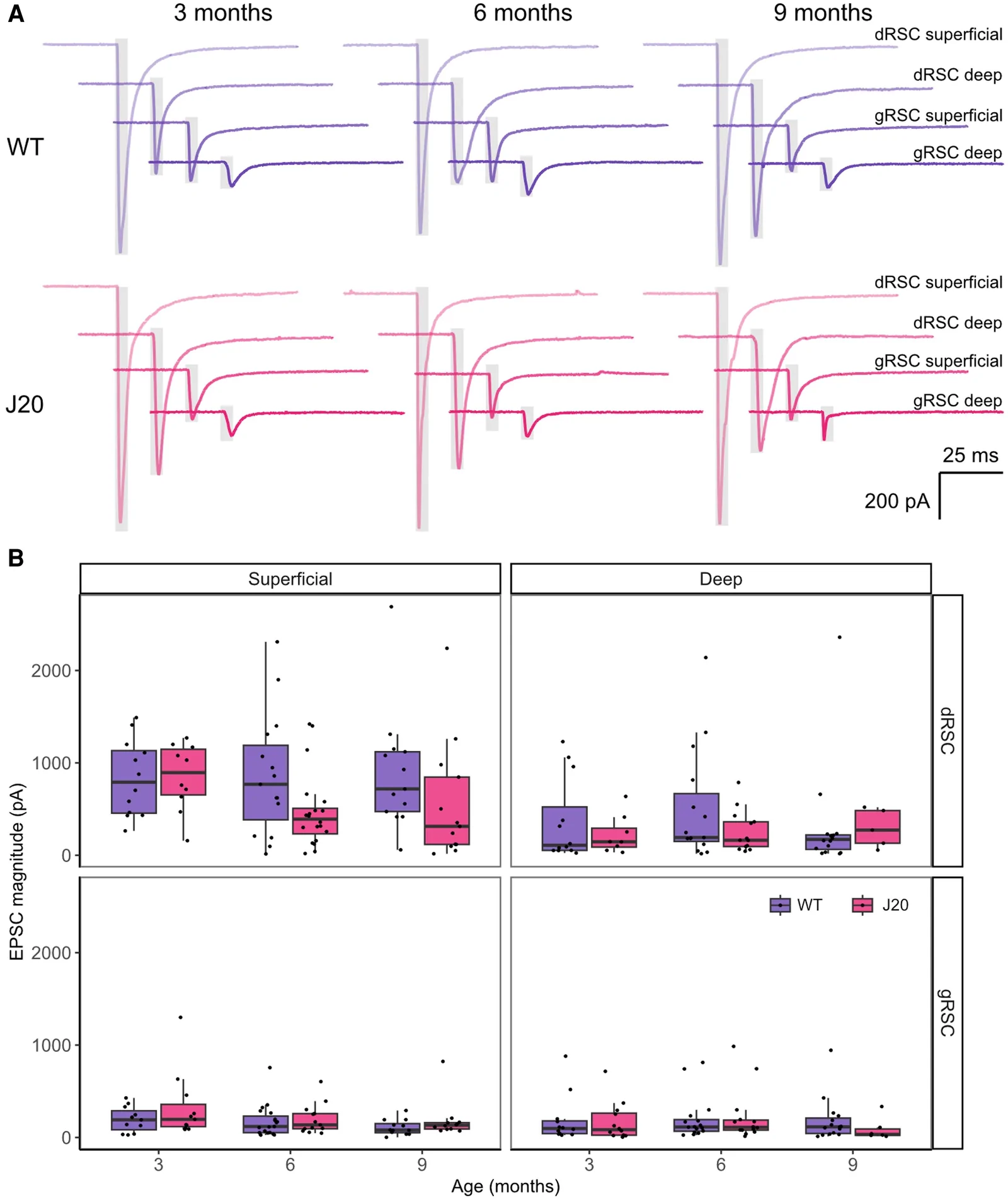
**EPSC magnitude differs between RSC sub-region and cortical layer, but is not affected by age or J20 genotype**. (**A**), Representative voltage-clamp (V_H_ = −70 mV) traces showing the first EPSC generated from a 7 Hz stimulation protocol in both dysgranular RSC (dRSC) and granular RSC (gRSC). Grey boxes indicate the 5 ms optical stimulation period (470 nm wavelength). (**B**), EPSC magnitude differed between RSC sub-region and cortical layer: magnitude appeared largest in the superficial layers of the dRSC, followed by the deep layers dRSC whilst gRSC superficial and deep layer EPSC magnitude appeared the smallest. Neither age nor genotype significantly explained variance in EPSC magnitude, and no clear difference in these factors are observed graphically. Boxplots display median (solid line), IQR and range. Statistical comparisons were made using linear mixed effects models, with the outputs from this model detailed in Table 1. Each data point represents one neuron, taken from both male and female mice.

**Table 2:** Fixed and random effect results for each mixed model analysing EPSC magnitude

	EPSC magnitude								
	Model 1			Model 2			Model 3		
Predictors	Estimates	*t*	*P*	Estimates	*t*	*P*	Estimates	t	*P*
Intercept	638.65	15.91	<0.001	724.35	11.43	<0.001	692.45	10.69	<0.001
	(559.64–717.65)			(599.62–849.08)			(564.93–819.96)		
Sub-region: gRSC	−351.77	−7.89	<0.001	−353.28	−7.95	<0.001	−353.87	−7.97	<0.001
	(−439.54–−264.01)			(−440.75–−265.80)			(−441.21–−266.52)		
Layer: deep	−206.14	−4.6	<0.001	−212.49	−4.74	<0.001	−214.87	−4.79	<0.001
	(−294.39–−117.89)			(−300.73–−124.24)			(−303.07–−126.67)		
Age: 6 months				−51.06	−0.81	0.421	−66.94	−1.07	0.285
				(−175.81–73.69)			(−189.89–56.01)		
Age: 9 months				−67.99	−1.01	0.313	−81.8	−1.24	0.216
				(−200.39–64.41)			(−211.74–48.14)		
Genotype: Tg				−90.72	−1.73	0.085	−91.67	−1.79	0.074
				(−193.95–12.52)			(−192.29–8.95)		
Sex: Female							87.36	1.7	0.089
							(−13.52–188.25)		
**Random effects**									
*σ* ^2^	146060.89			145540.38			145765.54		
*τ* ^2^_mouse_	13750.34			11751.37			9611.95		
ICC	0.09			0.07			0.06		
*N*	68			68			68		
Observations	305			305			305		
Marginal *R*^2^/Conditional *R*^2^	0.212/0.280			0.223/0.281			0.231/0.278		

For fixed effects (predictors), the Table displays effect size, confidence intervals, *t* statistic and significance value. For random effects, the Table displays residual variance (*σ*^2^), mouse variance (*τ*^2^) and the intra-class correlation coefficient (ICC). Marginal *R*^2^ refers to variance explained by fixed effects only, while conditional *R*^2^ refers to variance explained by combined fixed and random effects. There was also a random effect of mouse present in model 1, contributing 6.8% of variance to the total explained by the model, although the ICC score indicates a weak resemblance between mice. Each data point/observation represents one neuron with the breakdown by genotype, age and region detailed in Table 1. Both male and female mice were used: see Materials and methods for a full breakdown of sex by age and genotype.

We next analysed the time of onset of EPSC after optogenetic stimulation and the 20% to 80% rise time of the EPSC to infer whether amyloidopathy perturbed presynaptic release synchrony (Supplementary Fig. 4). Superficial pyramidal cells displayed a slightly faster EPSC onset than deeper pyramidal cells (Supplementary Fig. 4A and B), which would be expected due to ATN axons entering RSC in superficial layers.^39^ While modelling revealed that RSC subdivision, laminar location and sex had significant effects on the EPSC onset time, there was no effect of age or genotype with WT and hAPP-J20 mice displaying no differences (Supplementary Table 1). We found no effect of RSC subdivision or laminar location on 20–80% rise time, and subsequent models including age, genotype and sex did not improve upon the null model (Supplementary Fig. 4C and Supplementary Table 2).

Our final analysis of the AMPA-mediated component of the ATN-EPSC onto RSC pyramidal cells was to examine the paired pulse ratio (PPR) to determine whether hAPP overexpression led to changes in short-term synaptic plasticity (Supplementary Fig. 5) at different inter-stimulus intervals. As we reported previously,^39^ PPR varied significantly between dRSC and gRSC, as well as between deep and superficial layers, but we observed no effect of age or genotype (Supplementary Fig. 5 and Supplementary Table 3). Overall, we concluded that neither age up to 9 months nor the presence or absence of amyloidopathy had any significant effect on thalamo-retrosplenial signalling from the ATN.

### No change in NMDA/AMPA ratio in hAPP-J20 mice at any time point

Finally, we sought to determine whether the NMDA/AMPA ratio was altered in hAPP-J20 mice, as this is a sensitive measure of post-synaptic ionotropic glutamate receptor function and potential for plasticity at synapses. Example electrophysiological traces showing AMPA and NMDA-receptor mediated components of the ATN-EPSC are shown in Fig. 4 for each age, genotype and RSC subdivision. Again, we used a series of linear mixed effects models to determine the influences of these factors on the NMDA/AMPA ratio (Table 3).

**Figure 4:**
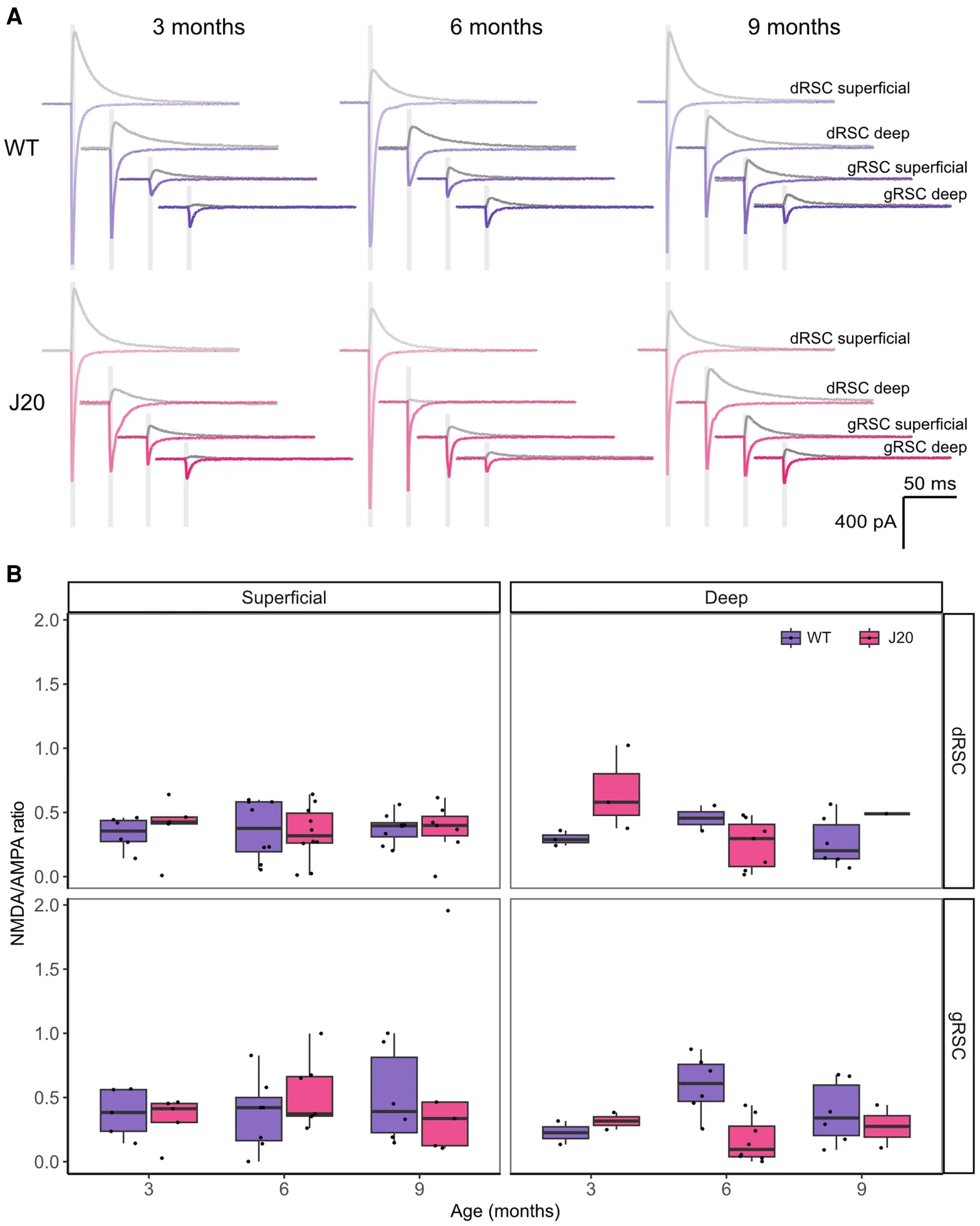
**NMDA/AMPA ratio does not differ between sub-region cortical layer, age or hAPP-J20 genotype**. (**A**), representative voltage-clamp traces showing AMPAR-mediated (blue/red trace) and NMDAR-mediated EPSCs (grey trace) generated by optogenetic stimulation (grey box indicates 5 ms optical stimulation period), in both dysgranular RSC (dRSC) and granular RSC (gRSC). AMPAR responses were recorded at V_H_ = −70 mV (standard aCSF) and NMDAR responses were recorded at V_H_ = +40 mV (standard aCSF containing 10 µM DNQX, 1 µM CGP55845 and 10 µM gabazine). (**B**), NMDA/AMPA ratio did not differ between sub-region, layer, age or genotype. Boxplots display median (solid line), IQR and range. Statistical comparisons were made using linear mixed effects models, with the outputs from this model detailed in Table 2. Each data point represents one neuron, taken from both male and female mice.

**Table 3:** Fixed and random effect results for each mixed model analysing NMDA/AMPA ratio

	NMDA/AMPA Ratio								
	Model 1			Model 2			Model 3		
Predictors	Estimates	*t*	*P*	Estimates	*t*	*P*	Estimates	*t*	*P*
Intercept	0.38	10.46	<0.001	0.38	7.12	<0.001	0.36	6.24	<0.001
	(0.31–0.45)			(0.27–0.48)			(0.24–0.47)		
Sub-region: gRSC	0.05	1.17	0.246						
	(−0.04–0.15)								
Layer: deep	−0.06	−1.3	0.195						
	(−0.16–0.03)								
Age: 6 months				0	−0.06	0.948	−0.01	−0.18	0.86
				(−0.12–0.11)			(−0.13–0.11)		
Age: 9 months				0.03	0.52	0.602	0.03	0.45	0.656
				(−0.09–0.16)			(−0.10–0.15)		
Genotype: Tg				−0.01	−0.23	0.818	−0.01	−0.22	0.827
				(−0.11–0.08)			(−0.10–0.08)		
Sex: Female							0.04	0.94	0.347
							(−0.05–0.14)		
Random effects									
*σ* ^2^	0.07			0.07			0.07		
*τ* ^2^_mouse_	0			0			0		
*N*	60			60			60		
Observations	127			127			127		
Marginal *R*^2^/Conditional *R*^2^	0.022/NA			0.005/NA			0.012/NA		

For fixed effects (predictors), the Table displays effect size, confidence intervals, *t* statistic and significance value. For random effects, the Table displays residual variance (*σ*^2^), mouse variance (*τ*^2^) and the ICC value. Marginal *R*^2^ refers to variance explained by fixed effects only, while conditional *R*^2^ refers to variance explained by combined fixed and random effects. Each data point/observation represents one neuron with the breakdown by genotype, age and region detailed in Table 1. Both male and female mice were used: see Materials and methods for a full breakdown of sex by age and genotype.

Analysis of NMDA/AMPA ratios found that model 1 (*fixed effects*: sub-region and layer) did not significantly improve upon the null model (*χ*^2^(2) = 2.8, *P* = 0.25; AIC_null_ = 28.9, AIC_M1_ = 30.2), and fixed effect estimates indicated no significant differences in NMDA/AMPA ratio between sub-regions and layers (Table 3). Model 2 (*fixed effects*: age and genotype) omitted area and layer as factors due to the lack of significance in model 1, but again did improve upon the null model (*χ*^2^(3) = 0.6, *P* = 0.89; AIC_M2_ = 34.3). Moreover, the addition of sex as a factor to model 3 did not significantly improve on model 2 (*χ*^2^(1) = 0.9, *P* = 0.35; AIC_M3_ = 35.4). Fixed effect estimates for age, genotype and sex in models 2 and 3 found no significant differences within these factors (Table 3). Finally, the null model found a negligible random effect of mouse on variance, which was also true in models 1–3 (Table 3 random effects). Therefore NMDA/AMPA ratio was not significantly affected by sub-region, layer, age or genotype (Fig. 4) and did not differ significantly between mice.

## Discussion

In this study, we set out to test the hypotheses that the RSC in hAPP-J20 mice would display evidence of the hypoactivity that is present in prodromal AD in humans,^8,9^ and that specific deficits in projections from the ATN to the RSC would be present in this model. However, we found no evidence to support either of these hypotheses and can conclude that signalling through ATN, at least to retrosplenial glutamatergic neurons, remains intact in the hAPP-J20 model, in contrast to deficits seen in areas such as hippocampus at similar time points in this model.^33,36-38^ This surprising lack of change in this circuit helps to explain why we observed no rescue of spatial memory when increasing the excitability of ATN using chemogenetics in the hAPP-J20 mouse model but could rescue novel object memory when targeting thalamic nucleus reuniens.^19^

### Basal c-Fos reactivity in the RSC

We found that the basal c-Fos reactivity was higher in dysgranular RSC compared with the granular subdivision. gRSC is associated with allocentric inputs arriving from dorsal subiculum,^41^ although we found that subicular inputs could similarly excite neurons in both dRSC and gRSC,^39^ while visual information associated with egocentric circuits preferentially arrives in dRSC.^41^ Given that the mice used for basal c-Fos were housed in their home cage before perfusion, being in a familiar environment without the need to navigate or execute task-specific behaviours may account for the lower c-Fos reactivity in gRSC, when dRSC would likely still be receiving input from the visual system irrespective of the behaviour of the mice.

Our observation that c-Fos expression in the RSC, CA1 and EC decreased with age is consistent with prior literature that describes decreased hippocampal and cortical basal levels of Fos protein and mRNA in aged rats.^42,43^ The mechanism behind this decrease is not clear, however, it may be linked to ageing-induced impairments in upstream signalling pathways such as MAPK,^44^ which is the primary pathway through which c-Fos expression is mediated.^45^ While the number of Fos^+^ cells decreased with age, there were no differences in basal c-Fos expression between WT and hAPP-J20 mice at 3, 6 or 9 months of age. This was an unexpected finding, as this marker of neuronal activity has been shown to be reduced in the RSC in another model of Aβ pathology.^16^ However, this reduction in c-Fos induction was observed following exposure to a novel environment. A different study using the APPswe/PS1ΔE9 model found reduced *Fos* mRNA expression in the CA1 region only after exploration of a novel environment but not in basal c-Fos levels.^46^ It may be that not using a behavioural spatial memory, navigation or exploration in our task failed to engage the RSC sufficiently to reveal any potential differences in RSC activity between WT and hAPP-J20 mice. Alternatively, there may just be no deficit present in this model at the time points that we measured.

### Synaptic properties of ATN projections to the RSC

We have previously found that both midline and anterior thalamic projections to cortical regions in the extended memory circuit elicit strong EPSCs, with both the nucleus reuniens^47^ and the ATN^39^ driving currents in the hundreds to thousands of pA range. Indeed, we previously found that the ATN formed monosynaptic connections with every pyramidal cell that we recorded from in RSC,^39^ and in the present study found every neuron out of the 305 that we recorded from received direct thalamic input. This study confirmed our previous findings^39^ that optogenetic stimulation of ATN axons drove larger EPSCs in dRSC than gRSC, and larger responses in superficial pyramidal cells.

However, we found that this projection did not change with age and appeared unaffected by amyloidopathy in the hAPP-J20 mouse model. We tested EPSC onset time, 20% to 80% rise time, PPR, AMPA and NMDA EPSC magnitude and AMPA/NMDA ratio. On every parameter measured, we found no difference in J20 mice relative to WT at any time point. We did not measure parameters such as presynaptic release probability or attempt to induce long-term synaptic plasticity, so it is possible that these aspects of ATN to RSC synaptic physiology are perturbed in J20 mice. It should be noted that this study targeted glutamatergic neurons in RSC only, and it is possible that thalamic input to GABAergic neurons in RSC may be perturbed in this model, given the disruption to inhibitory neuron signalling observed by others in the hAPP-J20 line.^38^

As discussed earlier, the RSC is particularly sensitive to deafferentation,^17,48^ and the ATN provide substantial glutamatergic input to this region. While the anterior thalamus does show amyloid pathology,^1,2^ we did not observe any amyloid plaque deposition in the ATN in hAPP-J20 mice at any time point tested. We hypothesized that Aβ oligomers present in the RSC would have synaptotoxic effects sufficient to disrupt this pathway, as is seen in other models and brain regions.^49-51^ However, our results indicate that post-synaptic Aβ pathology is not enough to impair synaptic responses and, therefore, suggests that disruption of the ATN to RSC pathway may not mediate cognitive deficits in pre-clinical and prodromal AD. This is supported by our recent findings that chemogenetic activation of the ATN does not improve behavioural deficits in J20 mice at 6m or 9m of age.^19^

### Retrosplenial cortex and ATN in amyloidopathy models

As we noted in the introduction, deficits in novelty-induced beta oscillations in the RSC can be observed in J20 mice by the time point that we investigated,^20^ a study carried out using the same colony as the present study. The study by Walsh and colleagues shows that electrophysiological deficits do occur in the RSC in J20 mice but our data show that this is unlikely to be driven by anterior thalamic projections to RSC. We previously found that a substantial portion of neurons throughout RSC receive input from both dorsal subiculum and anterior cingulate,^39^ but that the ATN provide the largest and most ubiquitous drive. Given the deficits in hippocampal function that others have observed in the J20 model and the importance of dorsal hippocampus in spatial memory, it is tempting to speculate that the deficits in beta bursting seen in J20 mice is due, at least in part, to disrupted hippocampal input to RSC. Lesions in ATN cause changes in GABAergic tone in *ex vivo* slices of rat RSC, along with the loss of the ability to induce long-term depression in the slices,^52^ so it is possible that the ATN provide a tonic excitatory input to RSC that is essential for maintaining a proper balance between excitation and inhibition. Perturbations to this system are possible in the J20 mice and would not have been detected using our experimental paradigm. It should also be noted that we did not observe any amyloid plaque deposition in the ATN at any time point measured in this study, which could also account for the lack of expected deficits in the ATN to RSC projection.

Despite our findings, one cannot exclude the possibility of the ATN to RSC pathway being perturbed in J20 mice. Deficits in anterior thalamic projections to GABAergic neurons or behaviour-specific changes to the ATN in J20 mice are possibilities that would be detected in our study. Future electrophysiological studies could interrogate subicular inputs to RSC in dementia mouse models, or perhaps study the integration of multiple inputs by RSC neurons. Perhaps deficits in projections from the ATN exist in AD, but only manifest in later stages of pathology (such as during periods of tau accumulation). The J20 mouse line models a relatively early AD pathology stage, with our chosen time points more akin to prodromal stages. It is also possible that the ATN inputs, with their large and ubiquitous drive onto RSC neurons, are somehow more resilient to AD-like pathology than other brain regions.

RSC afferent disruption may still be involved in cognitive impairment in AD, as pathways other than those with the thalamus could be affected in the early stages of the disease. One possible candidate is the dorsal subiculum input, which also exerts a strong excitatory influence on both subdivisions of the RSC.^39^ The dorsal subiculum contains a range of spatially-tuned cells and sends information on speed, trajectory and place to the RSC.^53^ Not only does the dorsal subiculum develop plaques at similar age-points to the RSC in hAPP-J20 mice,^54^ but lesioning this region also reduces the spread of Aβ pathology into the RSC.^55^ The RSC receives inputs from many brain regions, but it also sends efferent projections to widely distributed brain regions. Therefore, as the RSC is one of the first regions to show Aβ pathology in humans, perhaps its connectivity dysfunction lies in its efferent outputs. It is also possible that other mouse models of AD could show disrupted connectivity between the ATN and RSC, and that our chosen model was an unfortunate outlier for studying the cellular connectivity between these brain regions in dementia.

## Supplementary Material

fcag417_Supplementary_Data

## Data Availability

All data supporting the results are available within the manuscript or supplementary materials. The R code used to generate the graphs and analyses, along with all numerical raw data, can be downloaded from https://github.com/MickCraigLab/J20_ATN_RSC.
